# Development and content validity of the Functional Impacts of Narcolepsy Instrument: a novel patient-reported outcome measure for narcolepsy type 1 and type 2

**DOI:** 10.1093/sleep/zsag127

**Published:** 2026-05-13

**Authors:** Yulia Savva, Heather Romero, Stephen Crawford, Amy Howerter, Peter McPherson, Helen Doll, Tara Symonds, Todd Swick, Yves Dauvilliers

**Affiliations:** Global Medical Affairs – Neuroscience, Takeda Development Center Americas, Inc., Cambridge, MA, United States; Global Medical Affairs – Neuroscience, Takeda Development Center Americas, Inc., Cambridge, MA, United States; Global Medical Affairs – Neuroscience, Takeda Development Center Americas, Inc., Cambridge, MA, United States; Prospective Studies, Clinical Outcomes Solutions, Tucson, AZ, United States; Qualitative Science, Clinical Outcomes Solutions, Folkestone, Kent, United Kingdom; Quantitative Science, Clinical Outcomes Solutions, Folkestone, Kent, United Kingdom; Qualitative Science, Clinical Outcomes Solutions, Folkestone, Kent, United Kingdom; Global Medical Affairs – Neuroscience, Takeda Development Center Americas, Inc., Cambridge, MA, United States; Sleep-Wake Disorders Center, Department of Neurology, Gui-de-Chauliac Hospital, University of Montpellier, Montpellier, France; Institute of Neurosciences of Montpellier, INSERM, University of Montpellier, Montpellier, France; National Reference Network for Narcolepsy, Montpellier, France

**Keywords:** narcolepsy, NT1, NT2, patient-reported outcome, PRO, Functional Impacts of Narcolepsy Instrument, FINI, FINI-NT2, clinical outcome assessment, COA

## Abstract

**Study Objectives:**

Most patient-reported outcome measures for narcolepsy focus on excessive daytime sleepiness or do not focus on specific disease impacts (e.g. cataplexy, cognitive difficulties, fatigue, daily function). The Functional Impacts of Narcolepsy Instrument (FINI) was developed to measure key functional impacts in people with narcolepsy type 1 and 2 (NT1/NT2).

**Methods:**

The instrument was developed separately in NT1/NT2 populations with input from patients, clinical experts experienced in narcolepsy management, and clinical outcome scientists. Development included: patient experience interview studies informing item development; item development including de novo items, and modified/original items from the Patient-Reported Outcomes Measurement Information System (PROMIS) library; two waves of patient-debriefing interviews testing initial/modified item drafts; determination of scale structure using exploratory factor and Rasch analyses, and confirmatory factor analyses for NT2; consensus meetings between clinical sleep experts/clinical outcome experts to finalize the FINI draft items and structure with a 7-day recall period.

**Results:**

The final FINI for NT1 had 28 items within six independent domains: Tiredness, Cognitive Functioning, Cataplexy, Social Activities, Everyday Activities, and Everyday Responsibilities. The final NT2 version (FINI-NT2) included the same items excluding the Cataplexy domain (23 items). Final FINI and FINI-NT2 items demonstrated good content validity and covered the main impacts of NT1 and NT2, respectively, as confirmed by patient interviews, exploratory/confirmatory factor analyses, and Rasch analysis.

**Conclusions:**

The novel FINI assesses key functional impacts of narcolepsy in people with NT1/NT2 for use across clinical settings, and adds to the number of existing clinical outcome assessments for central disorders of hypersomnolence.

**Statement of Significance**

People with narcolepsy type 1 (NT1) or narcolepsy type 2 (NT2) often experience impacts on their daily life, such as cognitive difficulties, fatigue, and impaired work/school responsibilities, leading to decreased quality of life. Despite these recognized challenges, there remains a need for an additional tool that measures functional outcomes and complements existing narcolepsy symptom, severity, and impacts assessments. The Functional Impacts of Narcolepsy Instrument (FINI) was developed to evaluate functional impacts of narcolepsy that are important and meaningful to people living with NT1 (FINI) and NT2 (FINI-NT2). The instrument offers a patient-centered outcome measure for assessing narcolepsy impacts and treatment benefits related to changes in functional outcomes that can be used in both the clinical trial setting and clinical practice.

## Graphical Abstract

**Graphical Abstract:**
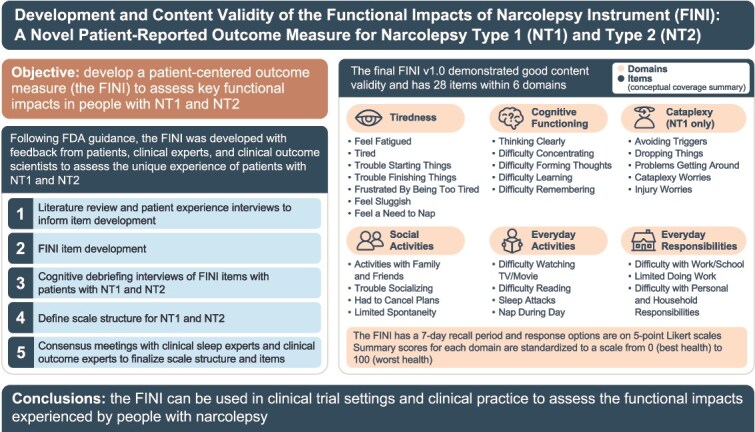

## Introduction

Narcolepsy type 1 (NT1) and narcolepsy type 2 (NT2) are chronic, rare, neurological central disorders of hypersomnolence (CDH) characterized by symptoms that disrupt sleep–wake regulation such as excessive daytime sleepiness (EDS), cataplexy (NT1 only), disturbed nighttime sleep, hypnagogic/hypnopompic hallucinations, and sleep paralysis [1–3]. Narcolepsy can impact an individual’s day-to-day function, including cognitive functioning and general activities of daily living, leading to decreased quality of life. It is also associated with mood disturbances such as anxiety and depression, and impacts on work and school performance, resulting in decreased work productivity and increased economic burden [4].

Symptom experience in people with narcolepsy has been recognized and reported over the last 2 decades [5–7]. The disease symptoms and daily functional impacts that matter most to people with narcolepsy gained increased attention as a result of the United States (US) Food and Drug Administration (FDA) Patient Focused Drug Development Initiative, which organized a Voice of the Patient (VoP) meeting on narcolepsy in September 2013 that included discussions with patients, patient advocates, and caretakers [8]. Consistent with the findings from the 2013 VoP meeting [8], the most commonly reported symptoms from a survey of people with narcolepsy (943 with cataplexy, 313 without cataplexy) were EDS (with cataplexy: 75.3%, without cataplexy: 87.5%), cognitive difficulties (46.9%, 53.0%), and general fatigue (40.4%, 54.0%) [9]. When survey participants were asked if narcolepsy affected their ability fully to perform activities important to them, the most debilitating impacts reported were on work or school performance (84.2%) [9].

Existing clinical outcome assessment (COA) instruments evaluating the symptoms or severity of outcomes associated with narcolepsy include the Epworth Sleepiness Scale (ESS) for assessing daytime sleepiness and a narcolepsy-specific instrument, the Narcolepsy Severity Scale for Clinical Trials (NSS-CT) [10–12]. The ESS is a patient-reported questionnaire commonly used as a primary endpoint in pharmacological investigations for narcolepsy and other sleep disorders such as obstructive sleep apnea [10], whereas the NSS-CT assesses a range of major narcolepsy symptoms [11, 12]. Other instruments used to evaluate impacts of disease in people with narcolepsy include the Patient-reported Outcome Measure for Central Disorders of Hypersomnolence (PROM-CDH) [13], Functional Outcomes of Sleep Questionnaire (FOSQ) [14], EuroQoL-5 Dimension 5-Level (EQ-5D-5L) [15], and Work Productivity and Activity Impairment (WPAI) questionnaire [16]. However, the EQ-5D-5L, WPAI, and FOSQ cover broader concepts (not narcolepsy-specific), or are specific to daytime sleepiness only. The PROM-CDH was the first to measure quality-of-life outcomes relevant to individuals with CDH, including five stand-alone items (daytime sleepiness, nap[s], driving a car, public transport, sexual activity), and five domains that measure the frequency of experiencing the following complaints and impacts: Outlook on Life; Energy, Attention, and Activities; Coping with CDH; Physical Well-being; Impact of Cataplexy. The FOSQ assesses the impact of daytime sleepiness on activities of daily living, the EQ-5D-5L is a health status instrument widely used in health economics, and the WPAI is used across different therapeutic areas to evaluate impacts on work productivity.

Thus, there is a need for a patient-reported outcome (PRO) instrument developed for narcolepsy that can more precisely capture and quantify specific aspects of the patient experience related to functional impacts of NT1 and NT2, particularly for use in clinical trials or to measure the impact of treatment in a clinical setting. COAs designed to assess concepts relevant to specific populations offer the most precise measurement of the potential impacts of interventions and are preferred by regulatory agencies for measuring pharmacological treatment benefit [17]. This study details the development of the Functional Impacts of Narcolepsy Instrument (FINI), a narcolepsy-specific PRO measure that complements existing COAs for CDH. An evaluation of the psychometric properties of the FINI has been conducted and will be presented in a separate publication. In the current manuscript, we present evidence supporting the content validity of this novel measure, which comprises both the FINI for people with NT1 and a modified version for people with NT2 (the FINI-NT2).

## Materials and methods

**Figure 1:**
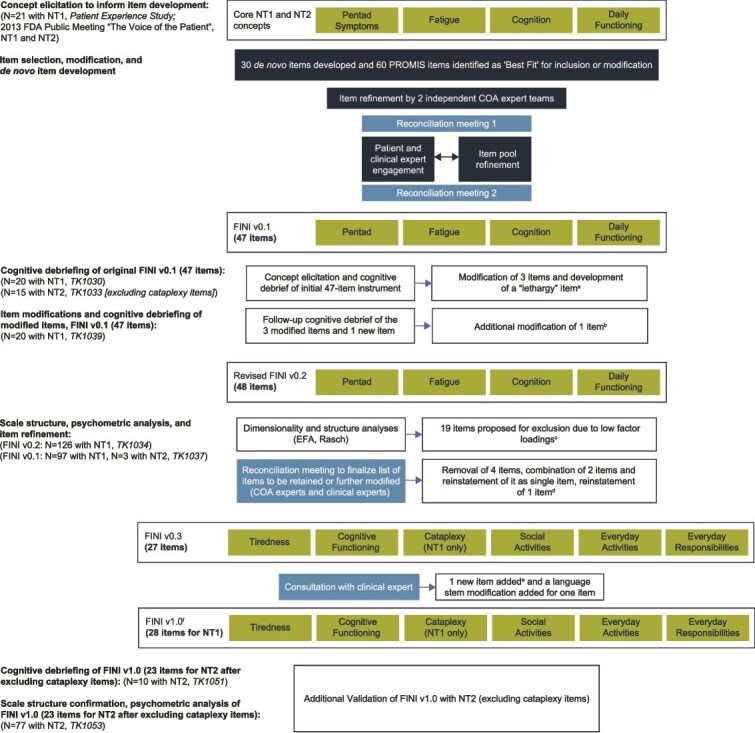
Development pathway of the FINI. COA, clinical outcome assessment; FINI, Functional Impacts of Narcolepsy Instrument; NT1, narcolepsy type 1; NT2, narcolepsy type 2; PRO, patient-reported outcome; PROMIS, Patient-Reported Outcomes Measurement Information System. ^a^Findings from patient cognitive debriefing studies of the FINI v0.1 (TK1030 and TK033) resulted in modifications of the following items: “Sleep Paralysis”, “Dropping Things”, and “Sleep Attack”. “Feel sluggish” was added given that the item “Listless” was not well-understood. ^b^Follow-up interviews (TK1039) resulted in further modification of the “Sleep Paralysis” item. ^c^EFA analysis resulted in 19 items with low factor loadings considered for exclusion (see Table 4 for the details). ^d^A reconciliation meeting resulted in the following: removing the “Sleep Paralysis”, “Weak All Over”, “Foggy Thinking”, and “Slow Thinking” items; reinstating the “Difficulty With Personal Responsibilities” and “Household Responsibility” items and combining them into a single item; reinstating the “Sleep Attacks” item. ^e^New item, “Feel a Need to Nap”, was added as supported by patient interviews and recommended by a clinical expert, and the “Sleep During Day” item wording was modified. ^f^Psychometric validation of the final FINI v1.0 was performed for a phase 3 study (N = 272 with NT1, TK1075).

An overview of the FINI development process is summarized in Figure 1. Development followed established FDA guidance for developing PROs [17]. For a summary of the studies used for FINI development and the key study inclusion criteria, see Table 1. Content validity was established with English speaking populations. After initial item selection, the English version of the FINI was translated in alignment with the International Society for Pharmacoeconomics and Outcomes Research *Principles of Good Practice for the Translation and Cultural Adaptation Process for PRO Measures,* which included concept elaboration (i.e. concept definitions that allow for harmonization across countries) [18], followed by dual forward translation, reconciliation, back translation, and review by local language experts. Items were cognitively debriefed with narcolepsy participants in the target language before finalization. During scale development, the FINI was translated into four languages: Spanish, Italian, Japanese, and Korean.

**Table 1:** Summary of the studies that supported FINI development

Study	Key study inclusion criteria	Development activity	FINI version
Patient Experience Study, NT1 *N* = 21	Adult (aged ≥18 years) Male or female Self- or physician-reported NT1 confirmed through both PSG and MSLT Self-reported cataplexy and EDS	CE interviews with adults with NT1 to understand participants’ experiences and develop an NT1-specific PRO questionnaire	FINI v0.1 (47 items)
TK1030 Content Validity Study, NT1 *N* = 20	Aged 18–65 years NT1 diagnosis via PSG or MSLT within the past 10 years ESS score ≥ 10 at screening History of cataplexy	Hybrid CE and CD interviews with adults with NT1 to explore content validity of the draft FINI and inform item modification	FINI v0.1 (47 items)
TK1033 Content Validity Study, NT2 *N* = 15	Aged 18–65 years NT2 diagnosis via PSG or MSLT within the past 10 years ESS score ≥ 10 at screening	Hybrid CE and CD interviews with adults with NT2 to explore content validity of the draft FINI and inform item modification	FINI v0.1 (42 items after excluding cataplexy items)
TK1039 Content Validity Study (Second Wave), NT1 *N* = 20	Aged 18–65 years NT1 diagnosis via PSG or MSLT within the past 10 years ESS score ≥ 10 at screening History of cataplexy	CD interviews with people with NT1 to test new and modified draft FINI v0.1 items (from studies TK1030 and TK1033), and CE discussions to understand participant experiences of living with cataplexy	FINI v0.1 (47 items)
TK1037 Phase 2 Narcolepsy Clinical Trial (*N* = 125); FINI was administered at baseline in participants with NT1 (*N* = 97) or NT2 (*N* = 3) (NCT04096560^*^)	Aged 18–65 years NT1 or NT2 diagnosis by PSG or MLST NT1 confirmed via HLA genotype ESS score ≥ 10 Discontinuation of all medications used for the treatment of NT1/NT2	Scale structure and psychometric properties of FINI in participants with NT1 or NT2	FINI v0.1 (47 items) FINI v0.3 (27 items)
TK1034 Standalone Observational Validation Study, NT1 *N* = 126	Aged ≥18 years NT1 diagnosis ESS score ≥ 10 at screening History of cataplexy	Scale structure and cross-sectional psychometric properties of FINI in NT1	FINI v0.2 (48 items) FINI v0.3 (27 items)
TK1051 Content Validity Study, NT2 *N* = 10	Aged 18–70 years NT2 diagnosis via PSG or MSLT within the past 10 years ESS score ≥ 10 in previous year	CD interviews with adults with NT2 to explore content validity of the FINI v1.0	FINI v1.0 (23 items for NT2)
TK1053 Standalone Observational Validation Study, NT2 *N* = 77	Aged ≥18 years ESS score ≥ 11 at screening NT2 diagnosis with no self-reported history of cataplexy No other medical disorder associated with EDS	Scale structure and measurement properties of FINI in NT2	FINI v1.0 (23 items for NT2)

CD, cognitive debriefing; CE, concept elicitation; COA, clinical outcome assessment; EDS, excessive daytime sleepiness; ESS, Epworth Sleepiness Scale; FINI, Functional Impacts of Narcolepsy Instrument; HLA, human leukocyte antigen; MLST, multiple sleep latency test; NT1, narcolepsy type 1; NT2, narcolepsy type 2; PRO, patient-reported outcome; PSG, polysomnography.

^*^Study of TAK-994-1501, an oral orexin receptor 2 agonist.

### Concept elicitation interviews

To inform item development of the FINI, concept elicitation (CE) interviews were conducted in people with NT1 in person or by telephone, using a comprehensive interview guide, to understand the relevant and meaningful symptoms and impacts of narcolepsy (Patient Experience Study, *N* = 21; Table 1). The interview questions were open-ended and designed for participants to speak about their experiences with NT1 and discuss concepts of interest deemed relevant to individuals with narcolepsy. These concepts were further explored in follow-up CE/cognitive debriefing (CD) studies (NT1: TK1030, *N* = 20, and TK1039, *N* = 20; NT2: TK1033, *N* = 15; Table 1).

All interviews were audio recorded and transcribed verbatim. NVivo v12 was utilized to assist with coding and analysis of qualitative data. CE interview data were analyzed using a thematic analysis approach whereby the researcher familiarized themselves with each transcript and noted initial ideas and then coded the transcript systematically. The researcher coded segments of text within the interview transcripts using a coding dictionary. Each segment of text was double-coded as either “spontaneous” or “probed”. The coding dictionary was developed before coding began, with standardized codes based on the interview guide, but was updated with new/revised codes as more transcripts were processed. To ensure quality, multiple coders were utilized. Initially, two coders independently coded a subset of transcripts; coding was then compared across these transcripts by reviewing the level of agreement between coders to ensure consistent application. Disagreements were then discussed and resolved.

The FDA VoP report (which covered a spectrum of symptoms across both NT1 and NT2) [8] and the CE study of NT1 were used to inform the key concepts meaningful and relevant to people with NT2. These concepts were explored further during the NT2 CE/CD interview study (TK1033, *N* = 15; Table 1).

Saturation analysis was conducted on CE data to examine if the most important symptom and impact concepts were elicited from the CE interviews (TK1030, NT1; TK1033, NT2) [19]. Transcripts were split into three sets based on the chronological order in which participants were interviewed. Concept saturation was achieved if a theme or concept emerged for the first time within the first two sets of interviews. If a theme/concept emerged only in the last set of interviews, and descriptions of this theme/concept suggested it may have been an important aspect of the patient experience, additional interviews may have been necessary to explore the concept further. However, in both studies, no new concepts were reported for the first time in the last set of interviews, thus saturation was deemed to have been achieved. The identified symptoms and impacts served as a basis for a preliminary conceptual framework of the FINI, which was subsequently finalized based on scale structure analyses (see details below).

### FINI draft items development

Both the FDA VoP report [8] and the multiple CE interview studies in participants with NT1 and NT2 informed item development of the FINI. The initial item pool was developed by two teams of COA experts and researchers working independently to select and draft the most relevant items for people with NT1 and NT2, and ensuring comprehensive coverage of the key conceptual themes identified during the qualitative studies. The initial pool included 60 items selected from the Patient-Reported Outcomes Measurement Information System (PROMIS) library and 30 newly developed items (90 items in total).

Patients were routinely engaged as part of the research team throughout the instrument development process. This included soliciting feedback on different iterations of draft items (including the final item set) from two patient advisors who were a part of a clinical trial study team, and vetting interim results of the Patient Experience Study in a US patient advisory board and with the patient/leader of a narcolepsy advocacy group in Europe. Finally, internationally recognized clinical experts in narcolepsy, who routinely treat patients and have been investigators in narcolepsy studies, provided consultation at different time points. Two of these clinical experts were particularly involved and were consulted on the draft items (Dr. Yves Dauvilliers [France] and Dr. Todd Swick [US]).

An initial reconciliation meeting with two teams of qualitative and quantitative COA experts was held to finalize the list of items to be retained and/or further modified (e.g. modified item stems, harmonized response categories). A second reconciliation meeting was held to align on the proposed final draft item set based on patient feedback from the Patient Experience Study, resulting in a 47-item pool for the FINI v0.1. The various iterations were recorded in an item-tracking matrix that detailed how the initial set of items were selected or modified, and how a small set of de novo items were created to arrive at the draft FINI v0.1 measure. All items in the FINI v0.1 draft measure went through additional exploration of conceptual coverage and comprehension in patient interviews (described below).

### Content validity of the FINI (CE/CD interviews)

Two interview waves were then conducted separately in people with NT1 and NT2 to confirm the retained items were content valid and covered the main impacts of NT1 and NT2 on patients’ lives. Both waves of interviews had a brief CE section and then focused on CD of the FINI. The CE section aimed to confirm that the FINI items had good conceptual coverage and that no new, unaddressed concepts emerged. The CD portion was completed with the goal of assessing item suitability, comprehension, and relevance, including evaluation of item stems, response options, and recall periods.

The first wave of interviews was conducted by telephone or web-based software in two separate studies with participants with NT1 (TK1030, FINI v0.1) and NT2 (TK1033, FINI v0.1 with excluded cataplexy items; Table 1). The interviews indicated that a few items needed modification to improve their comprehension and conceptual coverage. A second wave of interviews was conducted (separately for people with NT1 and NT2) to test the modified and new items proposed. For NT1, this was done with the same set of participants as the first interview wave (TK1039, FINI v0.1; Table 1). The results of this study led to the FINI v0.2 (48 items). The FINI v0.2 instrument was then carried forward to define scale structure. For NT2, the second wave of interviews was conducted later, after the scale structure was finalized (TK1051, *N* = 10, FINI v1.0 with excluded cataplexy items; Table 1).

### Scale structure of the FINI

The next step was to identify distinct domains that were meaningful to patients and to ensure that each domain accurately measured its intended construct. The scale structure was evaluated quantitatively using pooled data from an observational study of participants with NT1 (TK1034, *N* = 126, FINI v0.2; Table 1), and a randomized, placebo-controlled phase 2 study of participants primarily with NT1 (NCT04096560, TK1037; NT1: *N* = 97, NT2: *N* = 3, FINI v0.1; Table 1), providing a total of 226 participants. Responses were used to evaluate the dimensionality and scale structure of the draft FINI measure using exploratory factor analysis (EFA) followed by Rasch analysis. For EFA, items with pre-defined factor loadings ≥0.40 were considered for retention. Rasch analysis enabled the assessment of unidimensionality for each identified factor from the EFA based on model fit using the Van den Wollenberg Q1 Test (Q1), Akaike Information Criterion (AIC), Bayesian Information Criterion (BIC), and root mean square residual (RMSR) values.

### Finalization of the FINI scale structure

The evaluation of scale dimensionality led to 19 items being removed because of low factor loadings and poor model fit. Subsequently, a scale structure finalization meeting was held with a team of COA experts and a sleep clinical expert to review the results from the dimensionality and scale structure analysis and finalize the FINI items and domains, resulting in the FINI v0.3 (27 items). Additional modifications led to the final FINI v1.0 (28 items). These FINI item developmental steps were documented in the item tracking matrix.

### Confirmation of the FINI scale structure

During the development of the FINI instrument, an EFA was conducted because the structure of the FINI in NT1 was not yet known. After establishing the FINI structure in NT1, a confirmatory factor analysis (CFA) was conducted in NT2 to test whether the structure was held for the NT2 population. The scale structure confirmation for the NT2 version of the FINI was done using a stand-alone NT2 observational study (TK1053, N = 77, FINI v1.0; Table 1). Comparative fit index (CFI), non-normed fit index (NNFI), root mean square error of approximation (RMSEA), and standardized root mean square residual (SRMR) were reported as model fit statistics, where CFI >0.9, NNFI >0.9, RMSEA <0.08 (with an acceptance of higher values, up to 0.10, under certain contexts), and SRMR <0.08 indicated an acceptable model fit [20, 21].

## Results

### Participants

Demographic characteristics for participants of the studies used in the development of the FINI are summarized in Table 2. Clinical characteristics showed that most participants had moderate or severe symptoms. Among individuals with NT1, patient-reported severity was primarily moderate (70%, 14/20), with mild symptoms reported by 25% (5/20) of participants and very severe symptoms by 5% (1/20) (TK1030 study). In the NT2 group, most participants reported severe symptoms (53%, 8/15), followed by moderate (33%, 5/15) and very severe symptoms (13%, 2/15) (TK1033 study). For both populations, clinician ratings were consistent with patient-reported severity.

**Table 2:** Participant demographic characteristics of the studies utilized during FINI development

	Patient Experience Study NT1, *N* = 21	TK1030 NT1, *N* = 20	TK1033 NT2, *N* = 15	TK1039 NT1, *N* = 20	TK1037^*^ NT1, *N* = 97 NT2, *N* = 3	TK1034 NT1, *N* = 126	TK1051 NT2, *N* = 10	TK1053^†^ NT2, *N* = 77
Mean (SD) age, years	40 (11)	50 (12)	36 (10)	50 (12)	32 (10)	42 (15)	30 (10)	32 (8)
Sex, n (%) Female Male Prefer not to answer	14 (67) 6 (29) 1 (5)	13 (65) 7 (35)	12 (80) 3 (20)	13 (65) 7 (35)	75 (60) 50 (40)	87 (69) 38 (30) 1 (1)	6 (60) 4 (40)	68 (88) 9 (12)
Race, n (%) Caucasian/White Asian/Asian American Black/African American Hispanic American Indian/Alaska native Other/Multiple Prefer not to answer Not reported	14 (67) 0 1 (5) 4 (19) 0 1 (5) 1 (5) 0	15 (75) 0 4 (20) 1 (5) 0 0 0 0	12 (80) 2 (13) 0 0 0 1 (7) 0 0	15 (75) 0 4 (20) 1 (5) 0 0 0 0	71 (57) 28 (22) 16 (13) 0 0 2 (2) 0 8 (6)	104 (83) 0 10 (8) 0 0 12 (10) 0 0	8 (80) 0 2 (20) 0 0 0 0 0	73 (95) 4 (5) 3 (4) 0 2 (3) 1 (1) 0 0
Mean (SD) time since diagnosis, months	NA^‡^	54 (30)	63 (41)	54 (30)	NA	NA^§^	4 (3)	NA
Current treatment, n (%)	15 (71)	17 (85)	15 (100)	17 (85)	NA	NA^||^	10 (100)	75 (97)

FINI, Functional Impacts of Narcolepsy Instrument; NA, not available; NT1, narcolepsy type 1; NT2, narcolepsy type 2; SD, standard deviation.

See Table 1 for study descriptions.

^*^Baseline characteristics are summarized for the 125 participants in the phase 2 study.

^†^More than one selection was allowed for race.

^‡^Time range since diagnosis, years: 0.5 to 14.

§ Length of NT1 diagnosis (clinician-rated, *N* = 92), n (%): <1 year, 2 (2.2%); 1–5 years, 57 (62.0%); 6–10 years, 21 (22.8%); >11 years, 12 (13.0%); missing, 34 (participants recruited through the Patient Advocacy Group did not have clinician ratings).

|| Current medications for NT1 (multiple responses allowed): Provigil (modafinil), *n* = 37; Nuvigil (armodafinil), *n* = 21; Xywav/Xyrem (sodium oxybate), *n* = 8; Aptensio/Concerta/Ritalin (methylphenidate), *n* = 6; Sunosi (solriamfetol), *n* = 2; Wakix (pitolisant), *n* = 3; antidepressants, *n* = 41; Adderall/Dextroamphetamine/Vyvanse, *n* = 13.

### ce interviews

Key symptoms and impacts were elicited from interviews with participants with NT1 (Patient Experience Study; Table 3) and NT2 (TK1033; Table S1). For both populations, the most common symptom was EDS (NT1: *n* = 21 [100%]; NT2: *n* = 15 [100%]). Cognitive difficulties were also frequently reported (NT1: *n* = 18 [85.7%]; NT2: *n* = 15 [100%]).

**Table 3:** Key symptoms and impacts identified in concept elicitation interviews with individuals with NT1 (patient experience study, *N* = 21)

	Overall sample n (%)
Symptoms	
Excessive daytime sleepiness	21 (100.0)
Dozing off/falling asleep at inopportune times or places (e.g. events, talking to someone, sitting still, watching TV or a movie, reading, listening to someone talk)	21 (100.0)
Needing to take naps	18 (85.7)
Hard to get up in the morning/Not refreshed/Still sleepy upon waking	13 (61.9)
Falling asleep as safety hazard (e.g. while cooking/cutting something)	8 (38.1)
Actively seeks activities and jobs to stay more alert (e.g. high-paced job, keeping busy, getting up and walking around, takes breaks from boring things)	4 (19.0)
Cognitive difficulties	18 (85.7)
Issues with concentration, focus, attention	14 (66.7)
Brain fog^*^	12 (57.1)
Forgetfulness, problems remembering/recalling	12 (57.1)
Learning/processing issues	9 (42.9)
Impacts	
Independent/self-care	21 (100.0)
Decreased or no driving	20 (95.2)
Takes precautions/is cautious (e.g. safeguards while cooking, doesn’t do things alone/needs to have someone with them in case of emergency)	13 (61.9)
Financial hardship	10 (47.6)
Cannot or does not want to live alone (due to symptoms and/or medications)	5 (23.8)
Uses assistance to prevent falling (e.g. holding on to a cart or banister, using a cane)	5 (23.8)
Pays someone to help with things around the house (e.g. clean, deliver groceries, take care of yard)	2 (9.5)
Work/school	21 (100.0)
Stopped or decreased time working/in school	12 (57.1)
Receives accommodations/implemented strategies at work (e.g. breaks, naps, working earlier in the day, not working nights, needing flexible scheduling, avoiding triggers)	10 (47.6)
Hinders life goals (is not motivated to pursue dream career or change jobs)	8 (38.1)
Physical activities and health	20 (95.2)
Decreased or limited physical activity/exercise	18 (85.7)
Prone to collapsing and/or injury	18 (85.7)
Gained weight	13 (61.9)
Social and leisure activities	19 (90.5)
Decreased or limited participation in social and leisure activities (e.g. time with friends, going out, travel, hobbies)	18 (85.7)
Less travel	6 (28.6)
Actively avoids activities and situations that might trigger cataplexy attack (e.g. loud noises such as movies or fireworks, getting shocked/surprised, laughing in a social situation, hiking, dancing, amusement parks)	11 (52.4)
Dating is more difficult (does not date or has a harder time dating)	5 (23.8)
Mood and emotions	18 (85.7)
Depressed/unhappy/frustrated by not being able to do things; stays at home	14 (66.7)
Anxious/worried/embarrassed about symptoms interfering	13 (61.9)
Crabby/moody/cranky/impatient	7 (33.3)
Daily activities (e.g. household chores, grocery shopping, cleaning, laundry, cooking)	17 (81.0)
Nighttime sleep	14 (66.7)
Poor sleep quality	12 (57.1)
Wakes up during the night at night (unrelated to Xyrem [sodium oxybate] dozing)	10 (47.6)
Trouble falling back to sleep/tossing and turning/“Insomnia” (not being able to sleep when or as much as desired)	8 (38.1)
Vivid dreams	3 (14.3)
Family: time and activities together, roles and dynamics, intimacy	12 (57.1)
Quality of life	12 (57.1)
Other^†^	19 (90.5)
Drops things due to cataplexy	7 (33.3)
Improved lifestyle (e.g. eating healthier, watching diet, reducing stress, implementing coping strategies and positive changes)	6 (28.6)
Time management (e.g. not enough time to complete a task/not efficient; runs late)	5 (23.8)
Decreased or ceased drinking alcohol	4 (19.0)

EDS, excessive daytime sleepiness; NT1, narcolepsy type 1.

^*^Only a small number of participants used the term “brain fog,” with nearly all reporting it due to EDS. Most issues were related to memory/forgetfulness, concentration/focus, and feeling “fuzzy”.

^†^Examples of other impacts not listed (*n* ≤ 4 for each) include feeling different from others or “not normal,” being completely bedridden and unable to function due to EDS, needing to explain condition to others who may not understand/stigmatized, and exacerbation of other medical conditions.

Totals may exceed 100% as participants were allowed to report multiple impacts.

NT1 and NT2 significantly impacted various aspects of participants’ lives (Table 3 and Table S1). Participants reported impacts related to daytime sleepiness, cognitive difficulties, and daytime tiredness, as well as impacts on daily activities (e.g. decreased or no driving, household chores, inability to travel, cook or shower), and work or school (e.g. stopped/decreased work or school, change in work or career plans). Other impacts reported included on social and leisure activities and mood/emotions.

When discussing fatigue, energy, and tiredness, participants demonstrated a conceptual overlap among these experiences. In describing cognitive symptoms and their impacts, participants frequently mentioned difficulties with concentration, focus, memory, and learning. A small number of participants used terms like “brain fog” or “foggy” to describe a broader range of cognitive challenges, including issues with attention, concentration, and memory. This suggests that “brain fog” may serve as a catch-all term when participants struggle to distinguish between specific cognitive processes.

Participants with NT1 in the Patient Experience Study reported that cataplexy events generally happened in situations of high emotions or surprise, such as those associated with laughing, anxiety, or being upset, scared, or in shock (*n* = 12 [57%]). Less commonly reported triggers for cataplexy were physical exertion or tiredness, loud noises, and cold weather. Cataplexy most frequently impacted physical activities, often leading to falls, burns, and/or injuries, and participants reported that this symptom led them to avoid exercise and physical exertion, including work-related physical exertion.

The study achieved conceptual saturation. The identified symptoms and impacts informed the development of a preliminary conceptual framework of FINI, which comprised four domains: Narcolepsy Pentad, Fatigue, Cognition, and Daily Functioning [further divided into Work/School, Social, and Other].

### FINI draft items development

Based on the results of the CE study and the FDA VoP report, the FINI items were developed to reflect the four preliminary domains (Narcolepsy Pentad, Fatigue, Cognition, and Daily Functioning). From the initial pool of the identified 60 PROMIS items and 30 de novo items, a final set of 47 items was retained through a series of meetings with COA experts, the patient advisory board, and clinical experts.

The 47-item FINI v0.1 retained items from the PROMIS Short Form Fatigue 13a (five unmodified and one modified), and two modified items from PROMIS Item Bank v2.0—Cognitive Function (2016). The five items from the PROMIS Short Form Fatigue 13a were retained unmodified given their wide use across populations and the relevance of these items to people with narcolepsy. Although the PROMIS library served as a good basis for identifying content and items, other retained items of the FINI v0.1 were de novo items, particularly those in areas that were not covered by existing item banks or measures, but best capturing the impacts of NT1 or NT2 as described in the patient interviews.

All items used a five-point frequency scale from “never” to “always,” except for the items in the Cognition and Fatigue theoretical domains, which used a severity scale from “not at all” to “very much.” A 7-day recall period was used across all items.

### Content validity of the FINI (CE/CD interviews)

**Figure 2:**
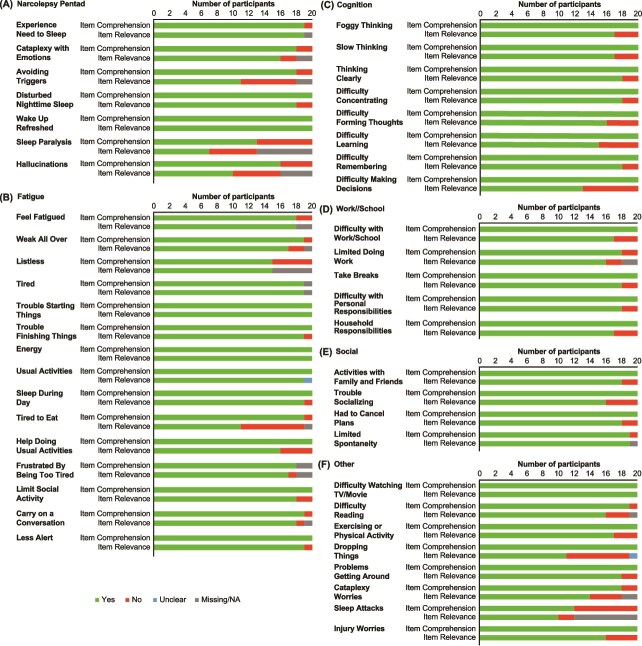
Summary of item comprehension and item relevance results from patient cognitive debriefing interviews of the FINI v0.1 in participants with NT1 (study TK1030; N = 20, wave 1 interviews). FINI, Functional Impacts of Narcolepsy Instrument; NT1, narcolepsy type 1. For a full version of the figure, see Figure S1. Missing: Participant did not answer the interview question or skipped the questionnaire item. NA: Participant not included in analysis as they did not understand concept or it was unclear if they understood concept.

After development of the draft FINI v0.1, content validity was confirmed in CE/CD interview studies in participants with NT1 (TK1030; Figure 2, Figure S1) and NT2 (TK1033; Figure S2). Most items were well-understood and highly relevant to the experiences of participants with NT1 and NT2.

For participants with NT1, most of the items (44 out of 47) were very well-understood, with at least 80% (*n* = 16) of participants demonstrating understanding of the item wording and concepts as intended (TK1030; Figure 2, Figure S1). Of these 44 items, two-thirds were understood by as many as 100% (*n* = 20) of participants. Some participants, however, demonstrated difficulty understanding three items related to sleep paralysis, feeling listless, and sleep attacks. These items were further modified or omitted during subsequent stages (see the next section for details).

Item relevance was only examined with participants who demonstrated an understanding of the item’s concept; most items were considered relevant based on participants’ experiences of NT1. Six items were flagged because fewer than 80% of participants considered them relevant to their experience of NT1. Two particular items (“Tired to Eat” and “Dropping Things”) were reported relevant by fewer than 40% of participants. Some participants felt the “Tired to Eat” item was more related to the ability to prepare or cook food; this item was eventually omitted in the next stages of development also because of a low EFA factor loading. The “Dropping Things” item was interpreted as a general problem, rather than relating to NT1. Thus, it was further modified (see the next section for details) but eventually omitted during the final reconciliation meeting.

CE/CD interviews with participants with NT2 (TK1033, FINI v0.1; Figure S2) indicated that 40 of the 42 questions (after removing cataplexy items) included in FINI v0.1 were very well-understood, with at least 80% (*n* = 11) of participants demonstrating a good understanding of the item wording and concept as intended. Eighty-six percent of items (36 of 42) were understood by all participants (*n* = 15). Two items, “Sleep Paralysis” and “Listless”, posed some comprehension difficulties among participants. These items were further modified or omitted (see the next section for details).

Most items were considered relevant based on participants’ experiences of NT2. However, 10 items were highlighted because they were considered relevant by fewer than 80% of participants who understood them. Of these items, “Tired to Eat” and “Injury Worries” were identified as not relevant by the highest proportion of participants (53% and 60%, respectively). “Tired to Eat” was eventually omitted in the next stages of development because of a low EFA factor loading. “Injury Worries” was omitted from the FINI version for NT2 given that the item was related to cataplexy and only relevant to people with NT1.

Both CE/CD studies demonstrated sufficient understanding and relevance of the proposed recall period (7 days), with only a small number of participants recommending a longer recall period for items such as learning new things and making decisions (NT2, TK1033) or limiting social activities and canceling plans (NT1, TK1030). Similarly, the response scales were acceptable to most participants; a small number of participants suggested including a “not applicable” response option for items related to work/school and social themes (NT1, TK1030; NT2, TK1033). Instructions to complete the questionnaire were well-understood by both populations.

The saturation of concepts was achieved separately in both the TK1030 (NT1) and TK1033 (NT2) studies, indicating that no new themes emerged beyond those already identified and covered by the novel instrument [19]. There was a moderate level of conceptual overlap in at least two items within each domain; further item reduction was thus considered and completed during the later stages of scale structure development (see next section).

### Modifications and CD interviews of FINI draft items

As a result of the interviews with participants with NT1 and NT2 conducted in the first wave, three items were modified, and a new item was developed. Then, the items were tested in participants with NT1 and NT2 separately in a second wave of interviews (NT1: TK1039; NT2: TK1051). The three items “Sleep Paralysis”, “Sleep Attacks”, and “Dropping Things” were modified to address participant concerns over item wording and to improve item understanding and conceptual relevance. The item “Feel Sluggish” was developed to capture participants’ experiences of feeling lethargic or sluggish. This item was included as the term “listless” and was not well-understood by most participants with NT1 and NT2. The rationale for modifying the three items is presented in Table S2.

The modified and new items were first tested in the second wave of CD interviews among participants with NT1. The results indicated that participants understood the modified “Sleep Attacks” and “Dropping Things” items. However, revisions to the “Sleep Paralysis” item did not lead to better understanding as the item was clear to just over half of participants. Some participants interpreted the item of “not being able to move” as physical weakness upon waking from nighttime sleep, rather than sleep paralysis. This may be tied to evidence suggesting that only about 25%–50% of narcolepsy patients report sleep paralysis [22].

Thus, it was decided to combine the wording used in the original and modified versions of the item on “sleep paralysis” to improve item clarity. This revised wording helped to ensure that those less familiar with the term “sleep paralysis” interpreted the item appropriately, as well as those who are more comfortable with the descriptive version. The item “Feel Sluggish” was well-understood and considered by 100% of participants with NT1 to be highly relevant.

For participants with NT2, the interviews were conducted on the items from the final version of the FINI instrument (FINI v1.0 excluding cataplexy items [23 items], TK1051; Figure S3). Overall, the FINI instructions were well-understood by participants, and no suggestions for change were provided. Additionally, all the items were well-understood by and considered relevant to the majority of participants. Of the 23 items, all items were well-understood and 19 items (82.6%) were considered relevant to those with NT2. Four items, however, had fewer than 80% of the participants considering them relevant. These items were: “Difficulty Forming Thoughts”, “Difficulty Learning”, “Trouble Socializing”, and “Limited Spontaneity”. Nevertheless, there were no issues with the response options, and the recall period was well-understood and found suitable by almost all participants; only one participant (10%) found the recall period challenging for the items in the Social Activities domain. The item “Feel Sluggish” was well-understood and considered by 90% of participants with NT2 to be highly relevant. Together, these analyses confirmed the content validity of the FINI items in participants with NT1 and NT2.

### Scale structure of FINI

EFA and Rasch analysis were utilized to define the FINI structure for a sample consisting of participants primarily with NT1 (pooled across TK1034 and TK1037). The EFA on the pooled data for participants primarily with NT1 supported a six-domain structure (Tiredness, Cognitive Functioning, Cataplexy [NT1 only], Social Activities, Everyday Activities, and Everyday Responsibilities; Figure 1). Additionally, the analysis indicated that 19 of the 48 items should be excluded based on factor loadings that were lower than the pre-specified criterion of 0.4 (Table 4).

**Table 4:** EFA factor loadings of the FINI (pooled data: study TK1034 [FINI v0.2] and study TK1037 [FINI v0.1])

	Factors					
Items	1	2	3	4	5	6
*Experience Need to Sleep*	*0.374*	*.*	*.*	*.*	*0.329*	*.*
*Cataplexy with Emotions*	*.*	*.*	*0.359*	*.*	*.*	*.*
Avoiding Triggers	.	.	0.432	.	.	.
*Disturbed Nighttime Sleep*	*.*	*.*	*.*	*.*	*.*	*.*
*Wake Up Refreshed*	*.*	*.*	*.*	*.*	*.*	*.*
Sleep Paralysis	.	.	0.504	.	.	.
*Hallucinations*	*.*	*.*	*0.341*	*.*	*.*	*.*
Feel Fatigued	0.560	.	.	.	.	.
Weak All Over	.	.	0.417	.	.	.
*Listless*	*0.359*	*.*	*.*	*.*	*.*	*.*
Tired	0.640	.	.	.	.	.
Trouble Starting Things	0.567	.	.	.	.	.
Trouble Finishing Things	0.447	.	.	.	.	.
*Energy*	*.*	*.*	*.*	*.*	*.*	*.*
*Usual Activities*	*.*	*.*	*.*	*.*	*.*	*.*
Sleep During Day	.	.	.	.	0.470	.
*Tired to Eat*	*.*	*.*	*.*	*.*	*.*	*.*
*Help Doing Usual Activities*	*.*	*.*	*.*	*.*	*.*	*.*
Frustrated by Being Too Tired	0.417	.	.	.	.	.
*Limit Social Activity*	*0.301*	*.*	*.*	*0.369*	*.*	*.*
*Carry on a Conversation*	*.*	*.*	*.*	*.*	*.*	*.*
*Less Alert*	*.*	*.*	*0.331*	*.*	*.*	*.*
Feel Sluggish	0.423	.	.	.	.	.
Foggy Thinking	.	0.478	.	.	.	.
Slow Thinking	.	0.581	.	.	.	.
Thinking Clearly	.	0.602	.	.	.	.
Difficulty Concentrating	.	0.466	.	.	.	.
Difficulty Forming Thoughts	.	0.556	.	.	.	.
Difficulty Learning	.	0.470	.	.	.	.
Difficulty Remembering	.	0.419	.	.	.	.
*Difficulty Making Decisions*	*.*	*0.334*	*.*	*.*	*.*	*.*
Difficulty with Work/School	.	.	.	.	.	0.481
Limited Doing Work	.	.	.	.	.	0.469
*Take Breaks*	*.*	*.*	*.*	*.*	*.*	*.*
*Difficulty with Personal Responsibilities*	*.*	*.*	*.*	*.*	*.*	*.*
*Household Responsibilities*	*.*	*.*	*.*	*.*	*.*	*.*
Activities with Family and Friends	.	.	.	0.663	.	.
Trouble Socializing	.	.	.	0.534	.	.
Had to Cancel Plans	.	.	.	0.453	.	.
Limited Spontaneity	.	.	.	0.446	.	.
Difficulty Watching TV/Movie	.	.	.	.	0.509	.
Difficulty Reading	.	.	.	.	0.481	.
*Exercising or Physical Activity*	*.*	*.*	*.*	*.*	*.*	*.*
Dropping Things	.	.	0.537	.	.	.
Problems Getting Around	.	.	0.572	.	.	.
Cataplexy Worries	.	.	0.584	.	.	.
*Sleep Attacks*	*.*	*.*	*.*	*.*	*0.346*	*.*
Injury Worries	.	.	0.621	.	.	.

EFA, exploratory factor analysis; FINI, Functional Impacts of Narcolepsy Instrument.

Italicized items have factor loadings <0.40; loadings <0.30 are considered trivial and thus were suppressed and were not presented. Factors: 1 = Tiredness; 2 = Cognitive Functioning; 3 = Cataplexy (NT1 only); 4 = Social Activities; 5 = Everyday Activities; and 6 = Everyday Responsibilities.

The multi-dimensional nature of the FINI v0.3 was further confirmed using a series of Rasch analyses conducted on the defined six domains (Table S3). The models evaluated in the Rasch analyses confirmed overall multidimensionality. For the unidimensional model (i.e. the full *N* = 28 item model), the Q1 test had a *p* < .0001 and high AIC, BIC, and RMSR values indicating that instrument fit statistic did not support a unidimensional scale to be presented as a single total score scale. The Q1 tests at the domain level were nonsignificant with smaller AIC, BIC, and RSMR, except for the Cataplexy domain, which demonstrated a minor deviation from the model expectations (partial credit model [PCM], *p* = .047), confirming multidimensionality of the FINI scale overall.

### Final instrument FINI v1.0

A meeting was held with COA experts and a sleep clinical expert, where the results of the patient interviews and the scale structure analyses were considered, and a final agreement on the FINI items and domains across both NT1 and NT2 indications was reached. This scale structure finalization meeting led to additional modifications including the removal of four items, the combination of two excluded items and their reinstatement as a single item, reinstatement of one additional item, modification of one item, and an addition of a new item, which resulted in a FINI v1.0 (28 items):

- The “Sleep Paralysis” item was removed because sleep paralysis may not be commonly experienced by people with NT1 and NT2 according to the literature [22] and the conducted CE/CD interviews.
- The “Weak All Over” item was excluded because, although the EFA loading was >0.40 on Factor 3 (Cataplexy), it differed from the other items in the domain. Specifically, it focused on a cataplexy symptom rather than cataplexy impacts, and also had a different set of response options (“not at all” to “very much”) compared with the other items in this domain (“never” to “always”).
- The “Foggy Thinking” and “Slow Thinking” items were dropped given the totality of evidence demonstrating that their content was covered by other similar items. The conceptual overlap was confirmed by the patient interview studies (TK1030 [NT1], TK1033 [NT2]) and subsequent quantitative work demonstrating a high correlation with other similar concepts (data not shown).
- The “Difficulty With Personal Responsibilities” and “Household Responsibilities” items were reinstated and combined to improve conceptual coverage by referring to both personal and household responsibilities in a single item.
- The “Sleep Attacks” item had a low factor loading (r = 0.35), but it was retained as it was considered conceptually meaningful and relevant based on patient interviews (TK1030 and TK1033). Additionally, the modifications to the language of the initial item improved its comprehension as indicated in follow-up interviews (TK1039 [NT1], TK1051 [NT2]).
- The “Sleep During Day” item was reworded based on input from a clinical expert: “sleep” was changed to “nap” to improve conceptual coverage of the symptom.
- An additional item was added to the Tiredness domain (“Feel a Need to Nap”), based on its relevance and meaningfulness to the concept of tiredness and fatigue, as supported by patient interview data and the input of a sleep clinical expert during reconciliation meetings. The new item was later debriefed with a similar population of participants with CDH, including NT2 (TK1051 study) and idiopathic hypersomnia, and was found to be relevant and well-understood. Across interviews, patients used “tired” or “low energy” to describe the state that resulted in needing a nap. Importantly, patients did not differentiate these states in the same manner that clinicians might. Instead, they indicated that the need to nap was a natural response to their tiredness. For instance, one individual reported “…I’m always tired a couple times during the day…I feel like I’m ready to fall asleep at any given moment…I still feel the need [to] take a nap during the day”. Because patient narratives consistently linked tiredness/fatigue with an increased need to nap, the item “Feel a Need to Nap” reflected a patient-expressed manifestation of tiredness, rather than a separate construct.

**Figure 3:**
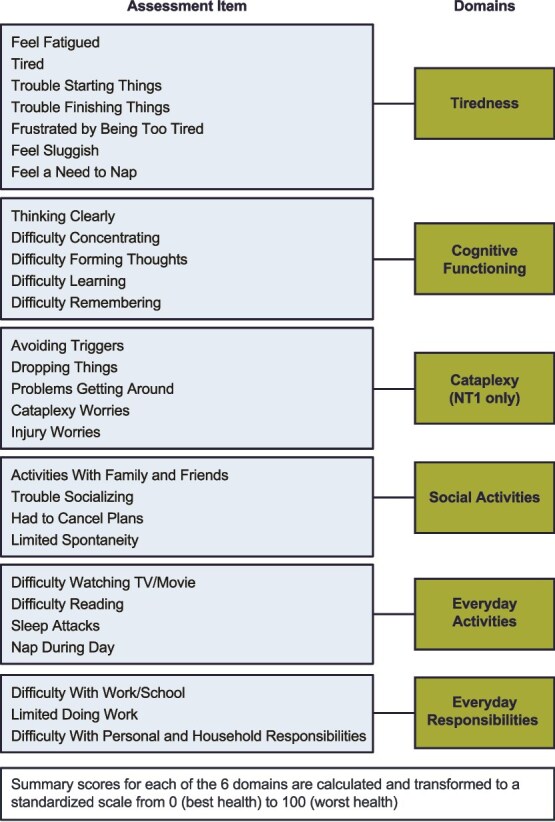
Conceptual framework of the FINI. FINI, Functional Impacts of Narcolepsy Instrument; NT1, narcolepsy type 1.

Based on the results of the scale structure analysis, the conceptual framework was finalized and updated to a six-domain structure. The FINI v1.0 includes six domains for NT1 and five domains for NT2 after excluding the cataplexy domain: Tiredness (Items 1 to 7), Cognitive Functioning (Items 8 to 12), Cataplexy (Items 13 to 17, NT1 only), Social Activities (Items 18 to 21), Everyday Activities (Items 22 to 25), and Everyday Responsibilities (Items 26 to 28). The recall period for the items is the “past 7 days”, and the response options are on five-point Likert scales. The final FINI v1.0 conceptual framework and the final 28 items for NT1 are shown in Figure 3. The final version for NT2 includes 23 items after excluding the five items in the Cataplexy domain.

The scoring algorithm was developed using a method similar to that of the European Organization for Research and Treatment of Cancer Core Quality of Life Questionnaire (EORTC QLQ-C30), involving a linear transformation to standardize raw scores on a 0-to-100 scale [23]. Similar to missing data rules applied to other PROs such as the EORTC QLQ-C30 [23] or the Short Form-36 Health Survey (SF-36) [24], the FINI domain scores can be calculated if at least half of the items in the domain have been answered (see the item numbers per each domain indicated above), otherwise the domain score is set to missing. Then, each domain score is averaged across the item scores and standardized to a 0-to-100 scale (as domain average score/4 × 100), where 0 indicates the best health and 100 indicates the worst health.

### Scale structure confirmation for NT2

The FINI structure was confirmed in participants with NT2 using the CFA in a stand-alone observational study (FINI v1.0, TK1053). The FINI content for NT2 is identical to the FINI for NT1, except it omits the five cataplexy-specific items. The overall domain structure was maintained for the NT2 population (CFI = 0.924, RMSEA = 0.114, SRMR = 0.106, and NNFI = 0.913). The final version of the FINI for individuals with NT2 will be referred to hereafter as the FINI-NT2.

## Discussion

The FINI and FINI-NT2 were developed specifically for use in people with NT1 and NT2, respectively. Development involved a rigorous process with several rounds of participant interviews, content development, and modification based on concepts meaningful and relevant to people with NT1 and NT2, as well as reconciliation meetings with clinical outcome experts and sleep clinical experts who have experience treating individuals with narcolepsy. It also included quantitative research using observational and longitudinal studies to analyze and validate the scale structure.

The instrument addressed key symptoms and impacts that people with NT1 and NT2 described based on interviews from the FDA VoP [8] and from CE and CE/CD patient experience interviews. The items were carefully developed using the language of the interviewed participants (English) whenever possible to reflect the meaningful symptoms and impacts they experienced. Outcomes identified from CE interviews with people with NT1 and NT2 were consistent with narcolepsy symptoms such as EDS, tiredness, cognitive complaints, and cataplexy (only for NT1), and narcolepsy impacts such as on social, work, and school-related activities as well as activities of daily living described in the literature [1, 2, 4, 9]. Two rounds of CD interviews further solidified the instrument’s conceptual coverage and content validity. The CD interviews confirmed item understanding, as well as the suitability of the recall period and response options.

Rasch analysis confirmed that almost all domains measured uniform constructs, and only the Cataplexy domain showed a minor deviation from Rasch model expectations (i.e. Q1 test for PCM *p* = .047), likely due to the heterogeneous clinical nature of the cataplexy symptoms and impacts in NT1. The decision on retaining the unmodified domain was based on the robust EFA loadings (≥0.417), confirmed content validity through cognitive debriefing with patients, and a negligible difference between observed and expected Rasch RMSR values (0.002). Given the totality of evidence, these results indicate that the Cataplexy domain remains a clinically relevant measure for the NT1 population.

The final version of the FINI v1.0 is structured as a 28-item multi-dimensional tool in NT1 with a six-domain scale structure determined by EFA and further confirmed through Rasch analysis and consultations with clinical experts. The current publication focuses on the development and exploration of the novel instrument, thus, the EFA has been used in NT1 for exploratory purposes to establish the scale structure, while CFA has been used in NT2 to evaluate the FINI scale structure for this second population of participants. A future publication will examine the external validation of scale structure (CFA) in participants with NT1 using phase 3 clinical trial data from a separate NT1 sample.

To test if the established scale structure holds in NT2, the CFA was conducted, confirming the five-domain FINI-NT2 structure (23 items, excluding the five cataplexy items). Although the CFI and NNFI achieved the commonly accepted thresholds, the RMSEA and SRMR were higher than optimal. This pattern is not unexpected given the small sample size (*n* = 77), as RMSEA is known to inflate under low sample size. We retained the original scale model to preserve theoretical coherence and to reduce the risk of overfitting. This decision was further supported by good factor loadings exceeding 0.50 across all domains and by patient input confirming strong content validity. It is recommended that the factor structure be confirmed in larger, more robust clinical trial datasets of participants with NT2 to determine whether the elevated RMSEA reflects sampling error associated with the small sample size or the heterogeneous nature of observational study data.

The FINI offers a unique conceptual framework validated through both patient and clinical expert input, and expands the existing library of instruments commonly used in clinical trials for narcolepsy, which primarily focus on sleep-specific scales such as the ESS [10], Stanford Sleepiness Scale [25], Insomnia Severity Index [26], and Pittsburgh Sleep Quality Index [27]. It also adds to the existing tools assessing impacts and quality of life, such as the FOSQ [14] and the PROM-CDH [13], and overall symptoms and impacts of narcolepsy (NSS-CT) [11, 12]. When development of the FINI was initiated, the PROM-CDH was not yet available, however both these instruments measure important aspects of narcolepsy (i.e. the PROM-CDH measures the impact of narcolepsy on quality of life, whereas the FINI measures specific functional impacts). The FINI is differentiated from other available PROs because all six domains focus specifically on functional impacts, capturing both the frequency and severity of these impacts. Furthermore, its development process makes the FINI sensitive as a treatment-response measure in clinical trials settings.

The strength of the FINI lies in its detailed assessment of six distinct aspects of narcolepsy disease impacts. A score is calculated for each domain, rather than as a total score for overall impact, allowing assessment of specific disease impacts. The estimated time for completion of the instrument is approximately 5 minutes, representing a minimal patient burden. Its robust content validity supports its suitability not only for clinical assessments but, more importantly, for use in clinical trials that require a rigorous validation process [17].

The instrument was developed through interviews with a US patient population and was subsequently translated into four languages with a full linguistic validation in the narcolepsy population of each country to ensure conceptual equivalence and cultural relevance. The translation process followed established COA linguistic validation guidance [18]. Additional translations have been completed for future use in clinical trials.

It should be noted that in the qualitative studies (including the patient experience study and the TK1030 patient CE/CD interview study), 71% to 85% of participants were actively receiving treatment at the time of their interview (Table 2), which is common for a real-world population. This high proportion of treated participants was primarily due to the inherent difficulties of enrolling an untreated narcolepsy population, which is rare in clinical settings. Additionally, there is evidence to support that treatment status did not substantially alter patients’ perception of the core disease manifestations. This is supported by the totality of evidence, which included the three patient interview studies (i.e. the patient experience, TK1030, and TK1033 studies), the FDA VoP report [8], and the established literature on narcolepsy [5–7, 9]. These studies demonstrated similar and convergent concepts, confirming their robustness and relevance of these key symptoms and impacts for the narcolepsy population despite the high representation of treated participants. More specifically, participants in the CE interviews (TK1030, TK1033) continued experiencing symptoms and impacts, as reflected in their ratings of symptom severity and impacts despite receiving treatments. Including treated participants ensures that the FINI items remain relevant to the actual population for whom the tool is intended.

A limitation of the study is that the final FINI and FINI-NT2 do not include the functional impacts of disturbed nighttime sleep or impacts of narcolepsy on driving. Regarding disturbed nighttime sleep, this was explored as part of the pentad symptoms of narcolepsy in initial item review, but was eliminated during the development process, in part due to the participants’ difficulty recalling functional impacts that occurred during the nighttime hours. Although the impact of narcolepsy on driving was mentioned in interviews, there is variability in the relevance of driving as shown in the literature, with up to 36% of untreated participants with NT1 and up to 62% of treated participants reporting “Not at All/Not too much” when asked about the impact of EDS on driving during routine clinical assessment [11]. In a global clinical trial setting, driving experiences may vary depending on an individual’s driving status as well as cultural and country-specific factors. Thus, including a driving-related item as part of an instrument during a clinical trial with a shorter observation period could introduce variability in capturing the overall patient experience. Nevertheless, accessing a driving-related impact remains important for characterizing the broader narcolepsy experience. US FDA guidance recommends objective measures such as simulated driving tests, actual motor vehicle tests, or reaction time/psychomotor function assessments as the preferred method for determining the extent or presence of driving impairment [28, 29]. PROs, including sleepiness scales or visual analog scales, may complement these objective measures by providing additional context.

We acknowledge that there is some conceptual overlap between tiredness and sleepiness. Accordingly, the FINI Tiredness domain item, “Feel a Need to Nap”, may reflect elements of both constructs. Although “napping” is often interpreted within the broader context of sleepiness, its inclusion in the Tiredness domain is supported by patient interviews and clinical expert feedback as a functional response to tiredness. This is also supported by the origin of this item as a modification of an item from a fatigue-specific construct (PROMIS SF Fatigue 13a). Further psychometric evaluation examining the relationship between the FINI Tiredness items and tiredness and sleepiness constructs will be reported separately.

More broadly, the multistep process followed during content development for the FINI and FINI-NT2 items resulted in a patient-centric PRO with evidence of good content validity. Additional evidence of the psychometric measurement properties of the FINI domains will be reported in a separate publication.

## Conclusions

The FINI and FINI-NT2 address a significant unmet need as a narcolepsy-specific measure to assess functional impacts experienced by people with narcolepsy on outcomes that are meaningful and important for patients across six independent domains: Tiredness, Cognitive Functioning, Cataplexy (NT1 only), Social Activities, Everyday Activities, and Everyday Responsibilities. The instrument adds to the existing COAs for CDH and may be applied to evaluate treatment responses in clinical studies and clinical practice of individuals with NT1 and NT2.

## Supplementary Material

FINI_tool_development_supplement_zsag127

## Data Availability

The data underlying this article are available in the article and in its online supplementary material.
