# Supplementary material for: Development and content validity of the Functional Impacts of Narcolepsy Instrument: a novel patient-reported outcome measure for narcolepsy type 1 and type 2
Source: Sleep. 2026 May 13;49(8):zsag127. doi: 10.1093/sleep/zsag127 (PMC13472893; doi:10.1093/sleep/zsag127)
Supplement: Supplementary file 1 [file fini_tool_development_supplement_zsag127.docx]

**Supplementary Materials**

**Development and Content Validity of the Functional Impacts of Narcolepsy Instrument (FINI): A Novel Patient-Reported Outcome Measure for Narcolepsy Type 1 (NT1) and Type 2 (NT2)**

Yulia Savva^1*^, Heather Romero^1*^, Stephen Crawford^1^, Amy Howerter^2^, Peter McPherson^3^, Helen Doll^3^, Tara Symonds^3†^, Todd Swick^1†^, Yves Dauvilliers^4,5,6^

Affiliations

^1^Takeda Development Center Americas, Inc., Cambridge, MA, USA

^2^Clinical Outcomes Solutions, Tucson, AZ, USA

^3^Clinical Outcomes Solutions, Folkstone, UK

^4^Sleep-Wake Disorders Center, Department of Neurology, Gui-de-Chauliac Hospital, University of Montpellier, Montpellier, France

^6^Institute of Neurosciences of Montpellier, INSERM, University of Montpellier, France ^7^National Reference Network for Narcolepsy, Montpellier, France

*Co-lead authors

^†^At the time the study was conducted

Corresponding author: Yulia Savva; 350 Massachusetts Avenue #5080H, Cambridge, MA 02139; Yulia.savva@takeda.com

**Figure S1**. Summary of Patient Cognitive Debriefing Interviews of the FINI v0.1 in participants with NT1 (Study TK1030; N=20, wave 1 interviews).

**Figure S2**. Summary of Patient Cognitive Debriefing Interviews of the FINI v0.1 in Participants With NT2 (Study TK1033; N=15, Wave 1 Interviews).

**Figure S3**. Summary of Participant Cognitive Debriefing Interviews of the FINI v1.0 for Participants With NT2 (Study TK1051; N=10, Wave 2 Interviews).

**Table S1**. Key Symptoms and Impacts Identified in Concept Elicitation Interviews With Individuals With NT2 (Study TK1033, N=15).

**Table S2**. Summary of Suggested Changes to Selected FINI v0.1 Items for Revisions (Following the TK1030 and TK1033 Study Results).

**Table S3**. Rasch Fit Statistics for the FINI v0.3 Domain Structure (Pooled Data: Study TK1034 and Study TK1037).

**Figure S1**. Summary of patient cognitive debriefing interviews of the FINI v0.1 in participants with NT1 (Study TK1030; N=20, wave 1 interviews).

FINI, Functional Impacts of Narcolepsy Instrument; NT1, narcolepsy type 1.

Missing: Participant did not answer the interview question or skipped the questionnaire item. NA: Participant not included in analysis as they did not understand concept or it was unclear if they understood concept.

**Figure S2.** Summary of Patient Cognitive Debriefing Interviews of the FINI v0.1 in Participants With NT2 (Study TK1033; N=15, Wave 1 Interviews).

FINI, Functional Impacts of Narcolepsy Instrument; NT2, narcolepsy type 2.

Cataplexy items were not administered. Missing: Participant did not answer the interview question or skipped the questionnaire item. NA: Participant not included in analysis as they did not understand concept or it was unclear if they understood concept.

Figure S3. Summary of Participant Cognitive Debriefing Interviews of the FINI v1.0 for Participants With NT2 (Study TK1051; N=10, Wave 2 Interviews).

FINI, Functional Impacts of Narcolepsy Instrument; NT2, narcolepsy type 2.

Cataplexy items were not administered. The response options for all domains were understood by all ten (100%) participants. Missing: Participant did not answer the interview question or skipped the questionnaire item. NA: Participant not included in analysis as they did not understand concept or it was unclear if they understood concept.

**Table S1**. Key Symptoms and Impacts Identified in Concept Elicitation Interviews With Individuals With NT2 (Study TK1033, N=15).

| Symptom Area | Symptom | n (%) |
| --- | --- | --- |
| Excessive Daytime Sleepiness |  | 15 (100.0) |
| Cognition |  | 15 (100.0) |
|  | Memory | 11 (73.3) |
|  | Processing and comprehension | 8 (53.3) |
|  | Concentration and attention | 8 (53.3) |
| Fatigue |  | 15 (100.0) |
|  | Tiredness | 10 (66.7) |
|  | Exhaustion | 7 (46.7) |
| Low Energy |  | 13 (86.7) |
| Disturbed Nighttime Sleep |  | 12 (80.0) |
| **Impact Area** | **Impact** | **n (%)** |
| Work and Social Function |  | 14 (93.3) |
| Mood and Emotions |  | 14 (93.3) |
|  | Low mood and poor self-esteem | 13 (86.7) |
|  | Frustration and anger | 6 (40.0) |
|  | Fear, anxiety, and worry | 4 (26.7) |
| Social Functioning |  | 13 (86.7) |
|  | Social relationships | 11 (73.3) |
|  | Social activities | 11 (73.3) |
| Household Chores and Daily Tasks |  | 13 (86.7) |
| Physical Tasks and Exercise |  | 9 (60.0) |
| Problems with Driving |  | 8 (53.3) |
| Financial Impacts |  | 4 (26.7) |

NT2, narcolepsy type 2.

Table S2. Summary of Suggested Changes to Selected FINI v0.1 Items for Revisions (Following the TK1030 and TK1033 Study Results).

| Item | Revision |
| --- | --- |
| Sleep Paralysis | This item was updated with a more patient-friendly description of “sleep paralysis”; a term that was misunderstood by some participants in the cognitive interviews. Seven subjects (35.0%) either did not understand or misunderstood “sleep paralysis.” For example, it was misinterpreted as “a form of interruption,” “stiffness,” or having to “get the body moving,” or “like neck cramps.” A new description was chosen to better reflect how participants described their experience in the interviews. |
| Feel Sluggish | A new item was added due to subjects stating that asking about feeling “listless” was unclear. In the interviews, n=5 (25.5%) subjects did not understand or misunderstood this concept. For example, 1 subject commented “listless means you're forgotten, like, you're off the list, like we're done with you.”  To address this, a new item was created to better reflect how subjects described their experiences: “Feel Sluggish”. For example, one subject reported, “Lethargic is a, is the best way to describe it…” and another commented “I don't want to be confused or lethargic or stuff like that…” The term “sluggish” was used by one subject to describe how he felt during the day due to lack of sleep “And that awake time was super sluggish…”. Both terms were used in the new item. |
| Dropping Things | This item was not relevant/unclear to 9 of the 20 participants.  The item was updated with the inclusion of a cataplexy attribution, using the patient-friendly terminology of “muscle weakness”. In the CD interviews, concerns around low relevance were noted. Although other items in the original version of the PRO measure were also found to have lower rates of relevance, the “Dropping Things” item was unique in that the CD interviews revealed that the problem was primarily related to attribution.  Specifically, 4 (20.0%) subjects interpreted “dropping things” as being associated with multiple other factors, unrelated to NT1 or cataplexy. Low rates of relevance for the other items did not appear to be an attribution issue per se. Therefore, it was agreed that adding an attribution to NT1 to the item wording would hopefully address this problem and improve the relevance of the concept. |
| Sleep Attacks | This item was updated with a more patient-friendly description of “sleep attacks,” which was a term that was misunderstood by some in the cognitive interviews. In the cognitive interviews, n=8 (40.0%) did not understand or misunderstood this concept. For example, n=4 referred to sleep paralysis rather than attacks, while others related it more to injuring or hurting themselves during their sleep.  In the CD interviews, subjects demonstrated a good understanding of the concepts described in the “Experience Need to Sleep” item, therefore, similar terminology was chosen for the “Sleep Attacks” item. |

CD, cognitive debriefing; FINI, Functional Impacts of Narcolepsy Instrument; NT1, narcolepsy type 1; PRO, patient-reported outcome.

Table S3. Rasch Fit Statistics for the FINI v0.3 Domain Structure (Pooled Data: Study TK1034 and Study TK1037).

| **Test for parallel ICCs/IRFs** | | | | | | | **RMSR** | |
| --- | --- | --- | --- | --- | --- | --- | --- | --- |
|  | **L-L X2** | **p-value** | **Van den Wollenberg Q1 test** | **Equal item discrimination** | **AIC** | **BIC** | **Obs** | **Exp** |
| **Derived factors** | | | | | | | | |
| **All 28 items** | 11720.7 | 0.597 | 565.4 (df=432) p<0.0001 | 256.1 (df=27) p<0.0001 | 12388.7 | 14574.0 | 0.810 | 0.811 |
| Tiredness (6 items) | 2003.0 | 0.553 | 90.4 (df=80) p=0.199 | 17.6 (5 df) p=0.007 | 2445.0 | 3552.4 | 0.588 | 0.585 |
| Cognitive Functioning  (5 items) | 1455.7 | 0.534 | 39.6 (df=56) p=0.952 | 6.05 (df=4) p=0.301 | 1821.7 | 2683.5 | 0.588 | 0.590 |
| Cataplexy  (5 items) | 1805.3 | 0.573 | 65.5 (df=48) p=0.047 | 14.7 (df=4) p=0.012 | 2179.3 | 3064.4 | 0.736 | 0.738 |
| Social Activities  (4 items) | 1025.4 | 0.544 | 33.4 (df=33) p=0.446 | 6.3 (df=3) p=0.178 | 1375.4 | 2156.1 | 0.529 | 0.531 |
| Everyday Activities  (4 items) | 1238.7 | 0.465 | 18.5 (df=33) p=0.980 | 3.0 (df=3) p=0.563 | 1644.7 | 2559.3 | 0.596 | 0.595 |
| Everyday Responsibilities  (4 items) | 1078.1 | 0.537 | 36.7 (df=33) p=0.303 | 6.4 (df=3) p=0.168 | 1430.1 | 2216.4 | 0.542 | 0.549 |

AIC, Akaike Information Criterion; BIC, Bayesian Information Criterion; Exp, expected; L-L X2, Log-Likelihood chi-squared test; FINI, Functional Impacts of Narcolepsy Instrument; ICC, item characteristic curves; IRF, item response functions; Obs, observed; RMSR, root mean square residual.

Results for the partial credit model are shown. Note: In this analysis, the “Difficulty with Personal Responsibilities” and “Household Responsibilities” items in the Everyday Responsibilities domain were used as stand-alone items before they were combined into a single item in the FINI v0.3.

**Supplementary Methods**

Scoring Algorithm for the FINI v1.0:

1. The FINI consists of six domains. The domain items are as follows:

Tiredness: Items 1-7

Cognitive Functioning: Items 8-12

Cataplexy: Items 13-17

Social Activities: Items 18-21

Everyday Activities: Items 22-25

Everyday Responsibilities: Items 26-28

2. For each participant, calculate the average score for each domain, *if at least half the items in the domain have been answered:*

(sum of responses in the domain / number of completed items in the domain)

3. Standardize the average score to a 0 – 100 scale:
   a. Standardized score = average score/4 x 100
   b. 0 indicates the best health and 100 the worst health. A decrease from baseline would thus indicate improved health.

For example, if the 4 items in one domain (e.g., Social Activities) had the responses 1, 2, 3, 2, the average score would be 2 (calculated as (1+2+3+2) / 4) and the standardized score would be 50 points (calculated as 2/4 x 100).

The same scoring algorithm is valid for the FINI-NT2 (excluding the Cataplexy domain)
